# ACE2‐Variants Indicate Potential SARS‐CoV‐2‐Susceptibility in Animals: A Molecular Dynamics Study

**DOI:** 10.1002/minf.202100031

**Published:** 2021-08-10

**Authors:** Szymon Pach, Trung Ngoc Nguyen, Jakob Trimpert, Dusan Kunec, Nikolaus Osterrieder, Gerhard Wolber

**Affiliations:** ^1^ Pharmaceutical and Medicinal Chemistry Institute of Pharmacy Freie Universität Berlin Königin-Luise-Str. 2–4 14195 Berlin Germany; ^2^ Institut für Virologie Freie Universität Berlin Robert-Von-Ostertag-Str. 7–13 14163 Berlin Germany; ^3^ Department of Infectious Diseases and Public Health Jocky Club College of Veterinary Medicine and Life Sciences City University of Hong Kong

**Keywords:** ACE2, Molecular dynamics, Molecular modeling, SARS-CoV-2, Spike

## Abstract

Severe Acute Respiratory Syndrome Coronavirus 2 (SARS‐CoV‐2) continues to be a global threat, causing millions of deaths worldwide. SARS‐CoV‐2 is an enveloped virus with spike (S) glycoproteins conferring binding to the host cell‘s angiotensin‐converting enzyme 2 (ACE2), which is critical for cellular entry. The host range of the virus extends well beyond humans and non‐human primates. Natural and experimental infections have confirmed the high susceptibility of cats, ferrets, and Syrian hamsters, whereas dogs, mice, rats, pigs, and chickens are refractory to SARS‐CoV‐2 infection. To investigate the underlying reason for the variable susceptibility observed in different species, we have developed molecular descriptors to efficiently analyse dynamic simulation models of complexes between SARS‐CoV‐2 S and ACE2. Our extensive analyses represent the first systematic structure‐based approach that allows predictions of species susceptibility to SARS‐CoV‐2 infection.

## Graphical abstract

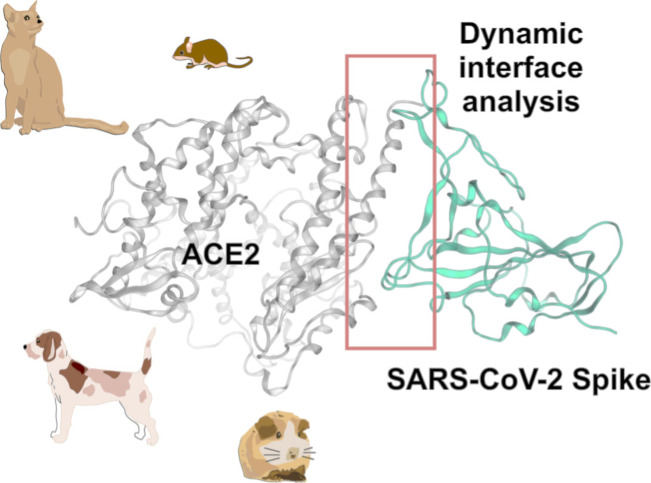

## 1 Introduction

Severe acute respiratory syndrome coronavirus 2 (SARS‐CoV‐2) emerged in 2019 and is responsible for the ongoing pandemic of the coronavirus disease 2019 (COVID‐19).^[1]^ As of January 2021, more than 100 million people have been infected and more than 2 million deaths have been recorded worldwide.^[2]^

SARS‐CoV‐2 and the related SARS‐CoV (SARS‐CoV‐1), which caused the outbreak of severe acute respiratory syndrome (SARS) in 2002–2004, are different strains of the species Severe acute respiratory syndrome‐related coronavirus of the family *Coronaviridae*. Coronaviruses (CoV) are enveloped viruses that present characteristic spike (S) glycoproteins on the surface of infectious virions that are essential for viral entry into a host cell.^[3]^

**Figure 1:**
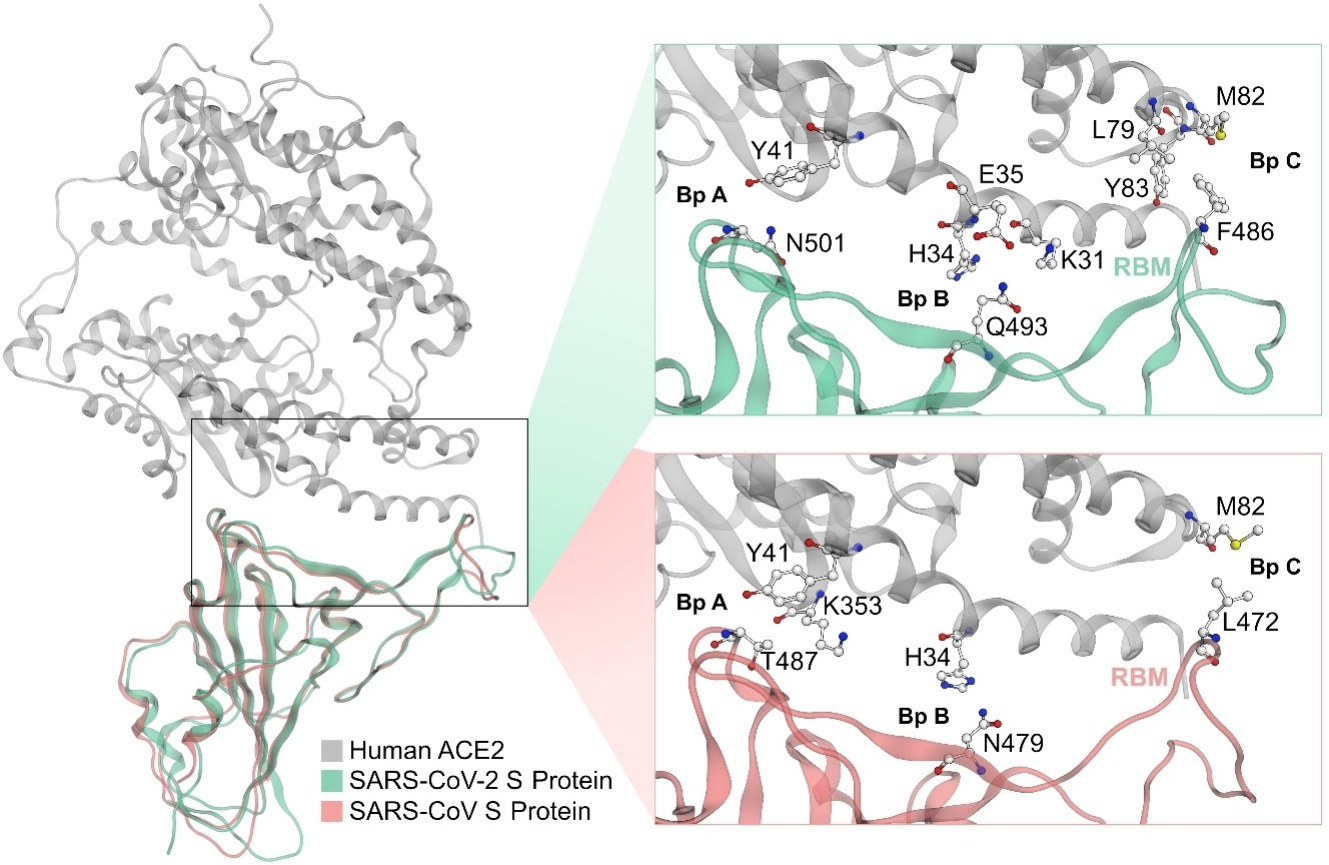
Three binding pockets (A, B and C) within the SARS‐CoV‐2 S protein – human ACE2 binding interface compared to the analogous binding interface of ACE2 with the SARS‐CoV S protein. Colour code: grey backbone – human ACE2, pink backbone – SARS‐CoV, green backbone – SARS‐CoV‐2.

S is a trimeric glycoprotein containing two functional subunits.^[4]^ The first subunit (S1) is responsible for binding to the host cell receptor, Angiotensin‐converting enzyme 2 (ACE2). The second subunit (S2) is responsible for the fusion of the viral and cellular membranes. The S1 subunit harbours the receptor‐binding domain (RBD), which is responsible for binding to host cells.^[3]^ The SARS‐CoV‐2 RBD forms an antiparallel β‐sheet structure, which is connected via short helices and loops.^[5]^ The contact to the host receptor is mainly established via loops known as the receptor‐binding motif (RBM, Figure 1). Compared to SARS‐CoV‐1, the RBM of SARS‐CoV‐2 appears to be less restricted in its conformation. This is because four out of five prolines present in a loop structure essential for binding to the ACE2 protein are replaced by more flexible residues such as glycine in the S glycoprotein of SARS‐CoV‐2.^[6]^

ACE2 is a membrane‐bound zinc‐metalloprotease expressed on the surface of cells not only in the respiratory tract but also in the heart, arteries, kidneys, and intestines, where it acts as a critical player in the endocrine renin‐angiotensin system. Independent of its enzymatic function, ACE2 is the cellular entry receptor for coronavirus infections. The SARS‐CoV‐2 S shows nanomolar binding affinity to this cellular receptor.^[7]^ ACE2 forms a big substrate‐binding cleft between its subdomains I and II.^[8]^ The SARS‐CoV S binds to ACE2 by exploiting a non‐competitive binding site on the outer side of subdomain I (Figure 1).

Crystal structures of the RBD of the S in complex with human ACE2 have been described for both SARS‐CoV^[9]^ and SARS‐CoV‐2.^[5]^ The studies have shown that binding interfaces contain three homologous binding regions (pockets) in the ACE2‐RBD interface, hereafter referred to as binding pocket A, B, and C (Bp A, B, C, Figure 1). The RBD of the SARS‐CoV S protein establishes hydrophobic contacts via T487 in Bp A to a lipophilic pocket formed by K353 and Y41 of ACE2.^[9]^ Moreover, hydrogen bonds are formed between N479 and ACE2‐residue H34 in Bp B, while lipophilic contacts are made between M82 (ACE2) and L472 in Bp C (Figure 1).

The SARS‐CoV‐2 S features a binding interface consisting of three contact regions that are homologous to SARS‐CoV. The binding interface is reported^[5]^ to utilize (i) hydrogen bonds in Bp A from N501 (homologous to T487 in SARS‐CoV1) to Y41 of ACE2, (ii) a hydrogen bond network via Q493 (homologous to N479 in SARS‐CoV) to ACE2‐residues K31, H34, E35 in Bp B, and (iii) lipophilic contacts in Bp C via F486 (homologous to L472 in SARS‐CoV) to ACE2‐L79, M82, and Y83 (Figure 1).

Recent reports confirmed that, in addition to primates, cats,^[10]^ ferrets,^[10]^ and Syrian hamsters^[11]^ are susceptible to SARS‐CoV‐2 infection, but pigs, chickens,^[10]^ mice and rats^[11]^ are not or, in the case of dogs, virtually asymptomatic and unable to transmit the virus.^[12]^ Studies on SARS‐CoV^[9]^ showed that differences in ACE2 sequences determine the variable susceptibility observed in different species. Based on these observations, we chose a structural approach to investigate how the genetic diversity of ACE2 orthologs may affect the susceptibility of animal species to SARS‐CoV‐2 infection. On an atomistic level, we have developed dynamic computational models for ACE2‐RBD complexes of different species allowing us to anticipate the effects of amino acid sequence variation of ACE2 on viral entry.

In parallel to our work, several groups have published their models^[13]^ primarily comparing ACE2 sequences from different animal species. However, these have either no or only limited structure‐based relevance. After our results had been published as a preprint in May 2020,^[14]^ Rodrigues et al.^[15]^ published a peer‐reviewed article in December 2020 reporting a static structure‐based approach that elucidates the contribution of ACE2 polymorphisms to SARS‐CoV‐2 susceptibility in different species. Their models allow simplified insights into S‐ACE2 interactions in different animal species and seem to confirm the results of our more comprehensive model.

Furthermore, several *in‐silico* studies on other SARS‐CoV‐2 proteins were performed (e. g. major protease),^[16]^ however without biochemical or biological validation of the results.

## 2 Computational Methods

### 2.1 Homology Modeling and ACE2‐SARS‐CoV‐2 S Models

Homology and loop models were prepared using MOE 2019 (Chemical Computing Group, Montreal, Canada). The sequences of animal ACE2 orthologs were aligned using the alignment tool integrated into MOE (Figure S1). The default MOE homology modelling workflow contains the following three steps: (1) initial partial geometry specification (keeping the template coordinates for identical positions and backbone coordinates for similar residues), (2) handling the insertions by modelling them as loops and deletions by removing residues from the template and energy minimizing the neighbouring residues, and (3) loop selection with side‐chain packing using a rotamer library containing side‐chain conformations from systematic clustering of molecular dynamics simulations. Finally, the models were refined and constructed using GB/VI scoring^[17]^ with a maximum of ten main chain models, default rotamer strain cutoff of 1.5 and distance cutoff of 1.2 Å, and automatic disulfide bond detection. ACE2 models of cat, dog, ferret, Syrian hamster, Chinese hamster, Campbell's dwarf hamster, and red squirrel were prepared using human ACE2 bound to RBD of SARS‐CoV‐2 (PDB‐ID: 6M0J^[5]^) with the best resolution as a template.

ACE2‐S complex models were prepared via superposition of receptor homology models onto the template crystal structure and removing human protein. The catalytic centre, as well as clashing side chains in the binding interface, were adjusted using a rotamer tool and the OPLS‐AA force field^[18]^ in MOE.

### 2.2 Molecular Dynamics Simulations

All systems were prepared in MOE 2019 by protonation^[19]^ (at 300 K and pH of 7) and capping SARS‐CoV‐2 S termini. ACE2 – RBD‐complex simulations were prepared with Maestro 11.7 (Schrödinger, LLC, New York, USA) and carried out with Desmond 5.5.^[20]^

All systems were inspected for atom clashes, optimized for H‐bonds, filled in a 12 Å large padding‐box with SPC water model,^[21]^ 0.15 M sodium chloride and sodium ions to keep isotonic and electrostatic neutral conditions. The systems were parametrized using the OPLS 2005 force field.^[22]^

The simulations were performed in five replicates per system under the default minimizing protocol. Briefly, the system was firstly simulated in a Brownian dynamics^[23]^ setup (at a temperature of 10 K over 100 ps), followed by a molecular dynamics (MD) simulation under NVT conditions (constant number of particles, volume, and temperature of 10 K) over 12 ps, restraining solute non‐hydrogen atoms. The final equilibration was performed under NPT conditions (with a constant number of particles, pressure, and temperature). Initially at 10 K over 12 ps with a fast temperature relaxation and slow pressure relaxation constant, followed by a temperature of 300 K over 24 ps. Finally, the non‐hydrogen atom restraints were removed and simulated over 24 ps under the temperature of 300 K and pressure of 1 atm with a fast temperature and normal pressure relaxation constant.

The simulations were conducted under periodic boundary conditions over 100 ns resulting in 2000 frames for each simulation. The main simulation runs were conducted as NPT ensembles. Nose‐Hoover thermostat^[24]^ and Martyna‐Tobias‐Klein^[25]^ were used to hold a constant temperature of 300 K and a pressure of 1.01325 bar, respectively. The electrostatic forces were explicitly calculated using a default distance limit of 9.0 Å. The integration of the forces was performed using the default RESPA integrator^[26]^ with a time‐step of 2 fs for bonded forces.

For visual inspection, all trajectories were wrapped and aligned onto backbone Cα atoms of the ACE2‐RBD‐complex and the first simulation frame using VMD 1.9.3.^[27]^

### 2.3 Trajectory Analysis

Initially, trajectories were analysed visually to find possible differences in the dynamic complexes, such as backbone movements. Further analysis was performed with Python 3.7^[28]^ using MDAnalysis 0.19.3 for the extraction of distances, angles, and hydrogen bonds from trajectories after an equilibration period of 10 ns (resulting in 1800 complex conformations per replicon). Data processing and transformation was done with pandas 0.25.3.^[29]^ Plots were created with seaborn 0.11.0^[30]^ and matplotlib 3.3.3.^[31]^ Principle component analysis was carried out with scikit‐learn 0.23.2.^[32]^ The numerical values of the eight molecular descriptors (depth of Bp C, deformation of Bp C, lipophilic contacts between S‐F486 and Bp C‐Y/F82/83, χ1 angle of ACE2‐Y/F82/83, distances S‐K417 – ACE2‐residue 29/30 and S‐Q493 – ACE2‐residues 30/31 and 34/35 in the Bp B, and hydrogen bond count in the Bp A) were standardized before the analysis using StandardScaler integrated into scikit‐learn. Each frame obtained from the MD simulations represents one instance (SARS‐CoV‐2 susceptible or not) of the data set.

## 3 Results and Discussion

### 3.1 Sequence Comparison of Animal ACE2 does not Explain Differences in COVID‐19 Susceptibility Between Investigated Species

We focused on animal species based on (i) their importance as natural reservoirs for SARS‐CoV‐2 due to frequent contact with humans and (ii) the availability of studies reporting susceptibility to SARS‐CoV‐2 infection enabling discrimination between differences in our models. The multiple sequence alignment of canine, feline and human ACE2 orthologs revealed that the residues reported as hotspots[^5^, ^9^] (referred to the human sequence positions 31, 34, 35, 41, 79, 82, and 83; canine positions are numbered one value lower than other species) show polymorphic mutations H34/33Y in dog and M82/81T in dog and cat. Since the difference in position 34/33 is present in dogs only, we searched for sequences harbouring the same polymorphism. We found that ACE2 of the common ferret (*Mustela pultorius*) also contains a tyrosine at this position.

However, ferrets can be infected with SARS‐CoV‐2,^[10]^ which suggests that the H34Y polymorphism does not prevent viral entry. Moreover, we compared ACE2 sequences from rodents including mouse, rat, Syrian hamster, Chinese hamster (*Cricteulus griseus*), Campbell's dwarf hamster (*Phodopus campbelli*) and red squirrel (*Sciurus vulgaris*) to sample additional binding pockets in the ACE2‐RBD interface and predict susceptibility to SARS‐CoV‐2 infection of the red squirrel, Chinese and Campbell's dwarf hamster.

Unfortunately, no protein structures were available for animal ACE2 at the beginning of the investigation. To compare three‐dimensional binding interfaces of animal ACE2‐RBD complexes, we developed homology models of dog, cat, ferret, Syrian hamster, mouse, rat, Chinese hamster, Campbell's dwarf hamster, and red squirrel proteins. We used the previously reported X‐ray crystal structure of human ACE2 bound to the SARS‐CoV‐2 RBD (PDB‐ID: 6M0J^[5]^) as a template. Due to high sequence identity between human and animal ACE2 orthologs (88 % for the Syrian hamster and red squirrel, 87 % for the Chinese hamster, 86 % for the cat and Campbell's dwarf hamster, 84 % for the dog, mouse, and rat, and 83 % for the ferret), all homology models show high quality, with no major deviations in backbone dihedral angles (a maximum of one Ramachandran outlier^[33]^ per model, Figure S2). Both outlier residues are located on flexible loops of ACE2 distal to the S binding site and represent polymorphic mutations from glycine in human crystal structure to serine in homology models.

Several months after our calculations, the cat and dog ACE2 cryoscopic electron microscopy (cryo‐EM) structures were published (PDB‐ID: 7C8D^[34]^ and 7E3J,^[35]^ respectively). Comparison of our homology models with the experimentally obtained structures revealed no significant differences, suggesting a high quality of our models. The backbone root mean square deviation between the homology models and the cryo‐EM structures amounts to 1 Å, indicating a correct global protein conformation. A carefully performed visual inspection of the S‐ACE2 interface revealed no significant differences in the conformations of the side chains involved in the protein‐protein binding.

All homology models show comparable flexibility in molecular dynamics (MD) simulations (measured as root mean square deviation of backbone heavy atoms) to the human enzyme in the range of 3–5 Å.

Since the homology models of animal ACE2 are based on coordinates of the human ACE2 in complex with the S RBD, we were able to superpose our models to the templates, yielding animal ACE2‐SARS‐CoV‐2 RBD complexes. All binding interfaces of animal ACE2‐S models remain in the same coordinate frame as the template crystal structure and thus comparable.

We compared ACE2 residues with direct contact to the RBD (distance of max. 4.5 Å) in all 3D complexes and searched for mutations possibly contributing to the low SARS‐CoV‐2 susceptibility observed in dogs. However, polymorphisms were present in both non‐susceptible and susceptible species, which did not explain the possibly lower affinity of RBD to dog, rat or mouse ACE2.

In the next step, we investigated the dynamic properties of the ACE2‐RBD complexes. All structures were simulated in five MD simulation replicas (100 ns each) and showed stable binding without dissociation events. For further analysis, we focused on three binding regions in the ACE2‐RBD interface.

### 3.2 Hot Spot Residue F486‐Binding to Bp C Depends on ‘Depth’ and Conformation of Bp C

We discovered that the RBM shows considerably larger movements for canine and rat ACE2 in complex with the RBD when compared to other species. We focused on the hotspot residue F486, which is situated at the top of the RBM and occupies the lipophilic pocket (Bp C) of ACE2.

**Figure 2:**
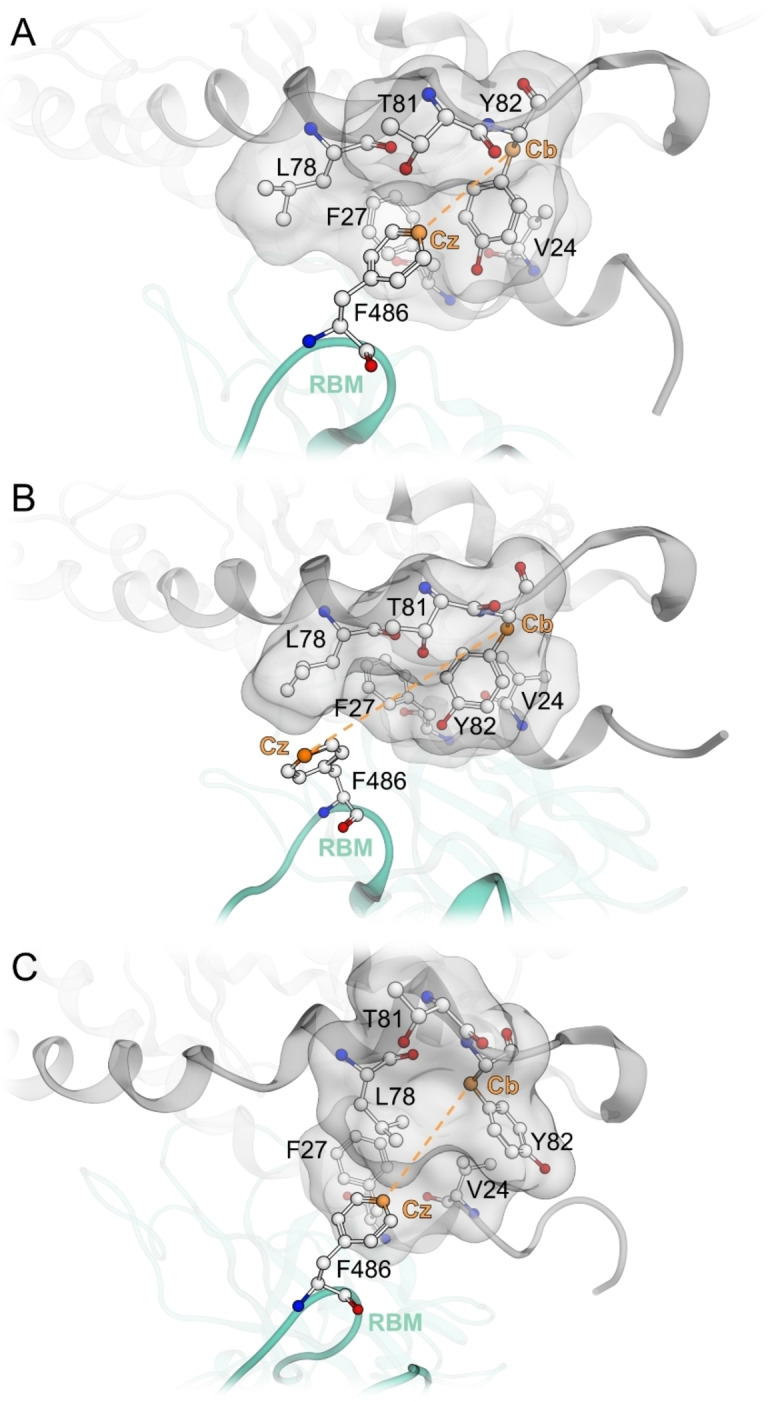
**A**: ‘Bound state’ of F486 in the distance of 5–7 Å (measured between F486‐Cz atom and Cb‐atom of Y82); **B**: ‘Fixed state’ of F486 outside the binding pocket; **C**: ‘Deformed state’ of Bp C. The numbering of residues refers to the canine sequence. Colour‐code: grey backbone – canine ACE2, green backbone – SARS‐CoV‐2, orange line – the measured distance between F486Cz and Y82Cb, grey surface – the molecular surface of canine ACE2 residues (V24, F27, L78, T81 and Y82).

**Figure 3:**
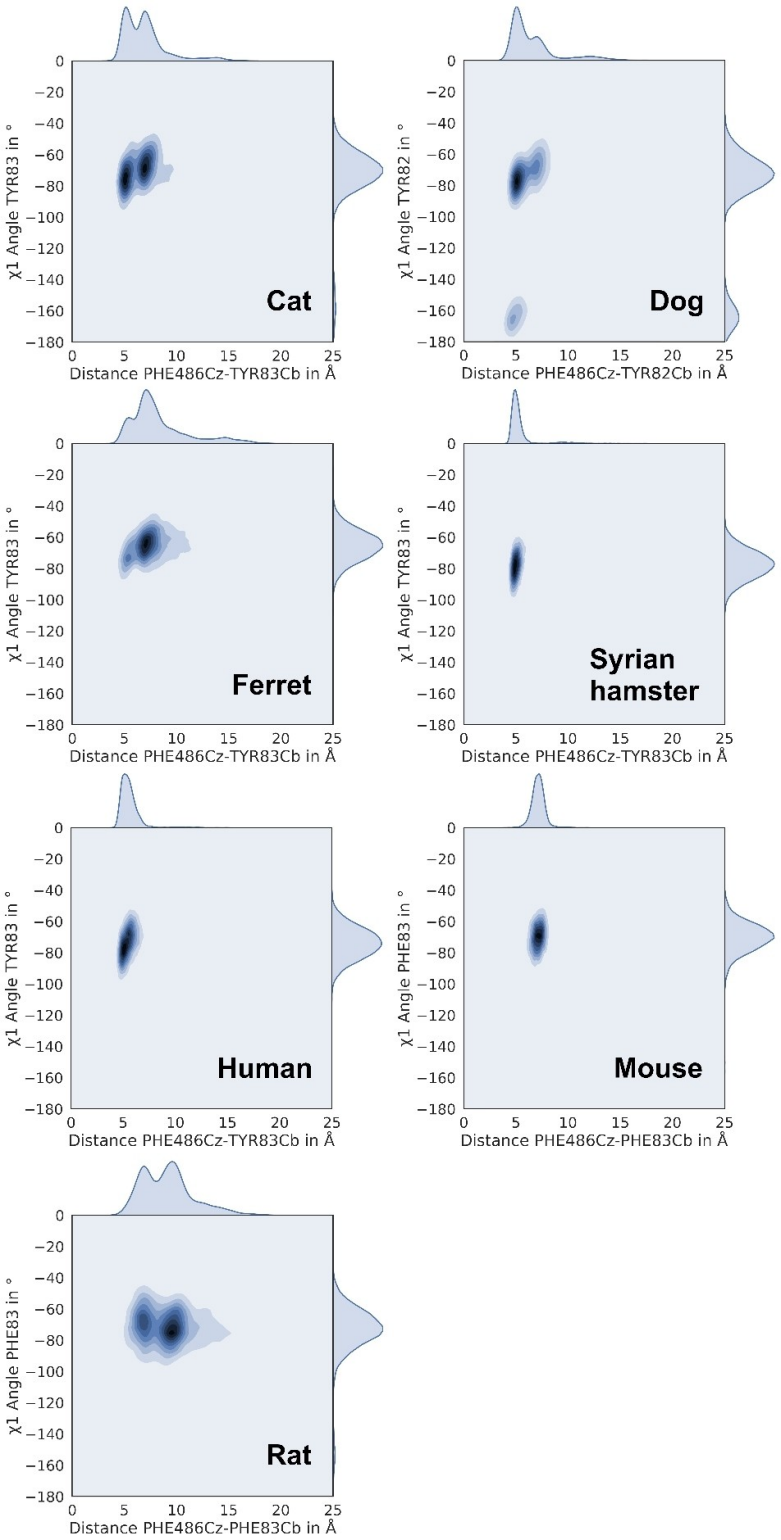
Occupation of binding pocket C indicated by kernel density plots (x‐axis: distance F486 Cz – Y83/82 Cb) and rotamers of central residue 83 (or 82 in dog).

We chose the distance between the Cz‐atom of F486 (position 4 on the phenyl ring) and the Cb‐atom of Bp C central residue 83 (or homologous position 82 in the dog model; Figure 2) as a surrogate parameter for F486 contacts. We observed three different states that can be adopted by the F486‐side chain: (i) a ‘perfect’ fit into the binding pocket (with a distance of ca. 5–7 Å, ‘bound state’, Figure 2A), (ii) contact with ACE2 outside the binding pocket, preferably with the lipophilic or aromatic side chain of residue 78/79 (with a distance of ca. 10–13 Å, ‘fixed state’, Figure 2B), and (iii) contact to lipophilic residues 27/28 and 78/79 with outwards rotated central Y82/83 in a ‘deformed state’ (with a distance of ca. 5–6 Å, Figure 2C). The contribution of the ‘fixed’ and ‘deformed state’ to RBD binding is yet unclear. Careful manual analysis of the performed MD simulations only revealed loose ACE2‐RBD F486 contacts in these states, which suggests negligible binding contributions. Of all analysed species, cat, ferret, Syrian hamster, human, and mouse showed one or two peaks with a narrow distance distribution around 5–7 Å, suggesting frequent occupation of Bp C (Figure 3). Although mouse ACE2 frequently occupies Bp C, we assume that weak interactions in the other two pockets (Bp A and B) might be responsible for the low SARS‐CoV‐2 susceptibility of mice. The simulations of rat ACE2‐RBD complexes showed a dominant peak at around 11 Å, implying F486 conformation in the fixed state. Canine ACE2‐RBD complex simulations show three peaks suggesting transitions between bound, fixed, and deformed F486 states. All these results (with exception of murine simulations) correlate well with the susceptibility of the species to SARS‐CoV‐2.

**Figure 4:**
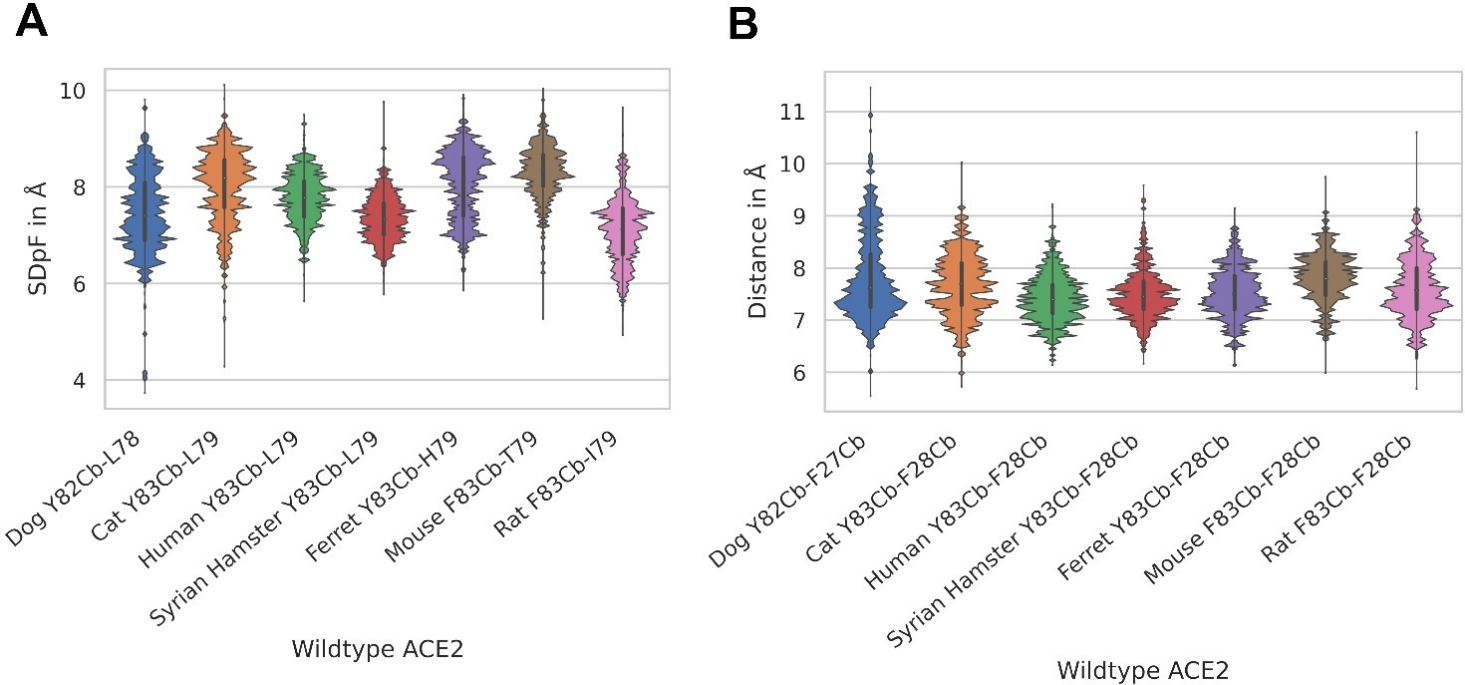
**A**: Distance distribution between the Cb atom of key residue 83 (or 82 in dog) and the side chain of residue 79 (or 78 in dog) flanking the binding pocket C representing the ‘depth’ of Bp C. **B**: Distance between the Cb atom of Y83 (or in dog 82) and the Cb atom of F28 (or 27 in dog) describing the deformation of Bp C.

We surmised that the conformational changes of F486 in dog and rat simulations might be caused by either flattening or conformational deformation of Bp C. To validate this hypothesis, we calculated distances between the Cb atom of central residue 83 (or 82 in the dog ACE2) as the deepest point of Bp C and all side chain atoms of residues at positions 24, 79 and 82 (dog homologues 23, 78 and 81) flanking this pocket. We plotted the occurrence of the shortest distance per frame (SDpF) for each residue (Figure 4A, S3). For all three descriptors, SDpF between residues 83–79 (or 82–78 in dog) correlates well with Bp C occupation in rat ACE2 (Figure 4A). The residue 83–79 SDpF average of rat (7.1 Å) is the lowest distance in comparison with other species, which indicates a flatter Bp C. This would result in a suboptimal fit into the binding pocket and entails unbinding events of residue F486. MD simulations revealed that the polymorphism I79L (rat – all other species) might be responsible for the narrowing of rat Bp C. We observed that the methyl group of I79 restricts the Bp C and hinders the binding of F486.

The canine ACE2 shows a broad SDpF distribution between residue 82–78 with a minimal distance of 6 Å, which is comparable to rat simulations. However, this state is less frequent in canine simulations. Additional χ1 angle measurements of epitope 83/82 show that this residue rotates out of Bp C, which causes a deformation of the binding pocket in canine ACE2. We chose the distance between Cb atoms of residues 83 and 28 (or 82 and 27 in dog) localized in the upper and lower helix, respectively, forming Bp C (Figure 4B). We surmised that the higher distance causes a larger shift between both helices and the resulting deformation of Bp C. In this state, the central residue Y83/82 can only establish weak interaction with F486. As expected, only canine ACE2‐RBD simulations show higher distances than 9 Å for the Y83/82‐F486 distance, which altogether suggests weak interactions. MD simulations revealed that the polymorphism V24/25 A (dog – all other species) might be responsible for the outward rotation of canine Y82. The larger and more rigid V24 pushes Y82 out of Bp C and towards the N‐terminus.

Subsequently, we analysed the differences in the distribution of residue 83–82 SDpF (or 82–81 SDpF in the dog, Figure S3). Syrian hamster, human, and rat show remarkably denser distance distributions (at approximately 5.5 Å) than other species. The sequence comparison shows that the three species express a long and non‐branched side chain residue (asparagine in Syrian hamster and rat, methionine in human) at position 82 (dog 81). These amino acids might stabilize F486 in the ‘bound state’ with Bp C by steric effects. The most favourable residue at position 82/81 for binding F486 seems to be the methionine present in human ACE2, which also increases lipophilic contacts at this position.

### 3.3 Mouse Mutation E/D30N and K31N in Bp B Disrupts Hydrogen Bond Network and Salt Bridges Found in Other Species

**Figure 5:**
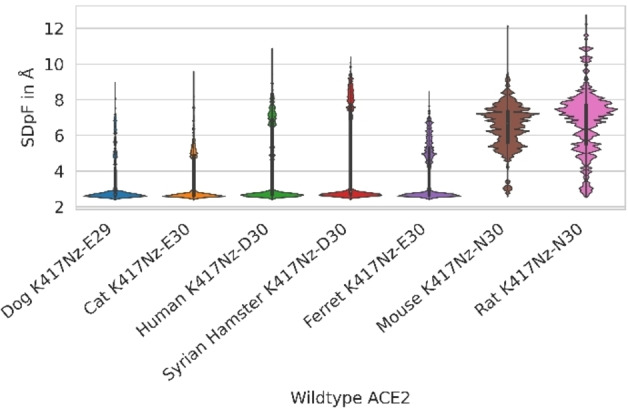
Shortest Distance per Frame (SDpF) between Nz atoms of RBD K417 and the side chain of ACE2 residue 30 (or 29 for dog) as surrogate parameters for interactions between these residues in the Binding pocket B for wild type ACE2.

**Figure 6:**
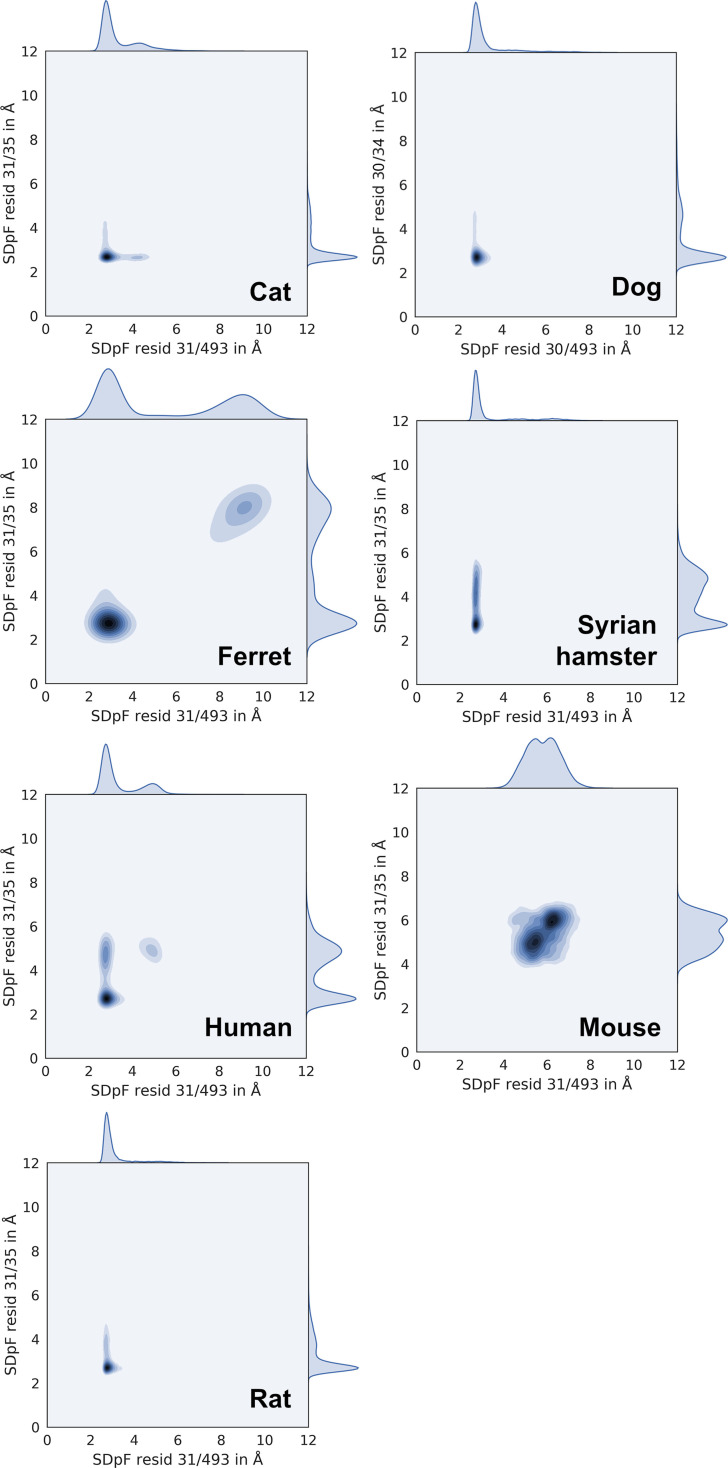
Interactions between ACE2 residue 31 and RBD residue Q493 summarized by kernel density plots (x‐axis, SDpF: Shortest Distance per Frame between Nz K31 or Cg N31 – side chain of Q493) and interactions between ACE2 residues 31 and 35 (y‐axis, surrogate parameter: distance Nz K31 or Cg N31 – side chain of E/D35).

Due to contradictory findings in murine Bp C models, we surmise that the low SARS‐CoV‐2 susceptibility observed in mice might be caused by unfavourable or less frequent interactions in Bp B and/or A. Hence, we searched for polymorphisms exclusively occurring in the mouse ACE2 sequence. We found two mutations in murine Bp B (E/D30N and K31N), which are surrounded by charged amino acids. Both mutations replace a charge with a neutral residue, suggesting that RBD stabilizing salt bridges cannot be formed. Further analysis of MD trajectories of susceptible species led us to the hypothesis that (i) residue E/D30 can interact with K417 of RBD and (ii) K31 interacts with E35 within the same helix of ACE2 introducing stable amino acid pairs coordinated by viral residue Q493. Our hypothesis is supported by distance (SDpF) measurements between residues 30 (ACE2) – 417 (RBD) (Figure 5) and 31 (ACE2) – 35 (ACE2)/31 (ACE2) – 493 (RBD) (Figure 6).

Simulations of the murine protein complex show a considerably greater distance between residues N31, E35 and Q493 compared to other species (6 versus 3 Å). This indicates disruption of the hydrogen bond network in Bp B. Similarly, the mutation E/D30 N occurring in mouse and rat breaks a salt bridge to K417 of RBD and the main distance peak occurs at 5–7 Å (in contrast to other species showing close contacts at ca. 3 Å). Due to larger distances for mouse and rat residues to RBD, water might invade the binding pocket resulting in lower S‐protein RBD affinity to murine ACE2.

In addition, we found that a serine residue occurs at position 27 in rat ACE2 while more lipophilic threonine epitopes are found in other species. Since residue 27 is surrounded by lipophilic residues of the RBD (F456 and Y489), we surmise that the T27S polymorphism in rat might contribute to less favourable interactions in Bp B.

### 3.4 ACE2 Position 353 Regulates Hydrogen Bonding in Bp A and Explains Missing Rat/Mouse COVID‐19 Susceptibility

**Figure 7:**
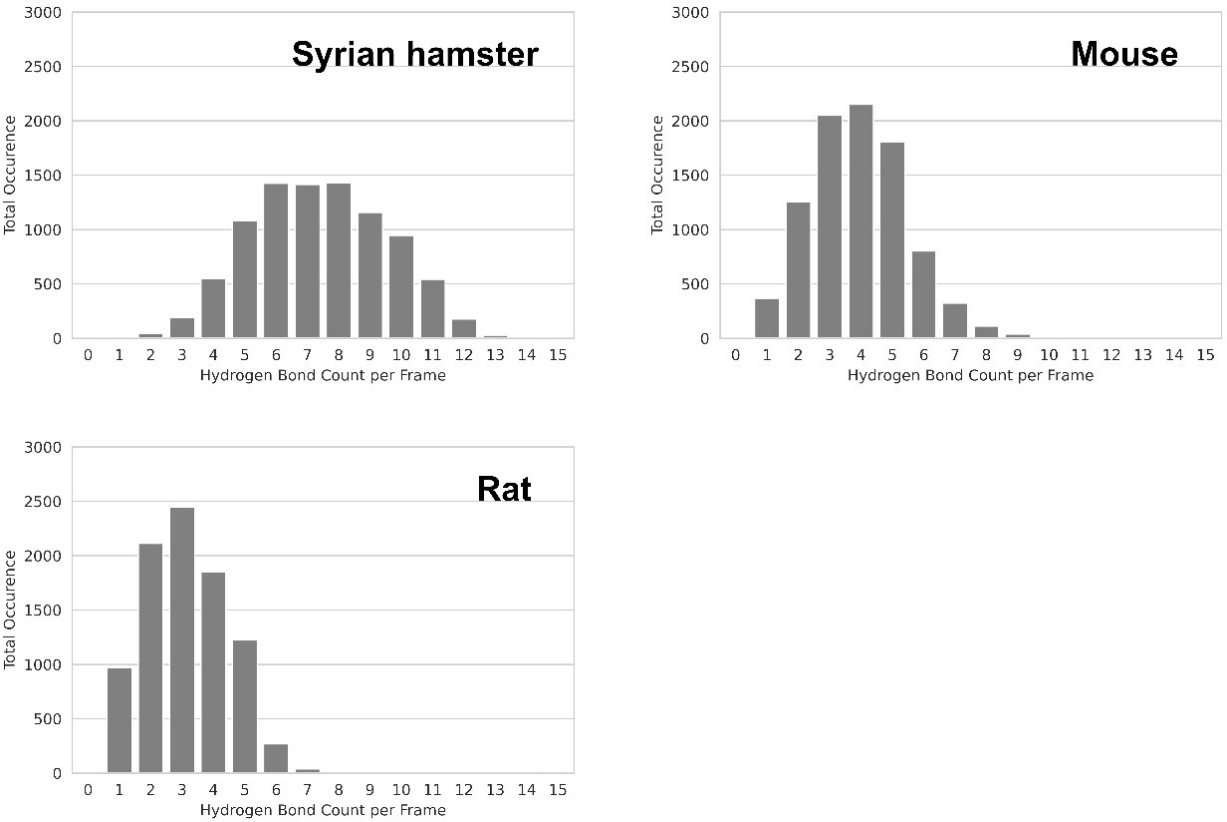
Total hydrogen bond count distribution in binding pocket A between ACE2 residues 37, 38, 41, 42, 353, 355 and RBD residues 449, 496, 498, 500, 501, 505 for Syrian hamster, mouse, and rat simulations.

To our knowledge, the role of residues involved in interactions within Bp A could not be clarified so far.^[6]^ We, therefore, compared the ACE2 sequence of rat and mouse with other species and identified the mutation K353H as a relevant difference. Inspecting MD trajectories of rodent ACE2‐RBD complexes, we observed that a lysine side chain in Syrian hamster ACE2 establishes a salt bridge to D38. Similar to the residue pair K31–E35 in the Bp B, which is coordinated by viral residue Q493, the pair K353‐D38 interacts with RBD residue Q498. This region of Bp A is surrounded by a hydrogen bond network consisting of ACE2 residues 37, 41, 42, 355 and RBD residues 449, 496, 500, 501, 505. We assume that, according to the O‐ring theory,^[36]^ this hydrogen bond network ‘seals’ the hotspot K353/D38 (ACE2) – Q498 (RBD) and prevents water from invading the protein‐protein interface. The histidine at position 353 in mouse and rat decreases the average number of hydrogen bonds established in the Bp A (7 in Syrian hamster versus 3 or 4 in rat or mouse, respectively; Figure 7).

Since histidine is less basic than a lysine, the formation of a salt bridge to D38 is less likely. MD simulations show that the histidine residue is too short to establish hydrogen bonds with D38 (ACE2) or Q498 (RBD). D38 can rotate out of the Bp A and water can reach the binding site, which results in less frequent interactions between the two proteins. This could explain why the RBD shows a lower affinity to mouse and rat ACE2.

### 3.5 Principle Component Analysis of Molecular Descriptors suggests Major Contribution of Bp B and C to the SARS‐CoV‐2 Susceptibility

To confirm the statistical relevance of the molecular descriptors characterizing the S‐ACE2 contacts for the species SARS‐CoV‐2 susceptibility, we performed a principle component analysis (PCA) as described in the Methods Section. The analyzed feature set comprises the depth of Bp C, deformation of Bp C, lipophilic contacts between S‐F486 and Bp C‐Y/F82/83, χ1 angle of ACE2‐Y/F82/83, distances S‐K417 – ACE2‐residue 29/30 and S‐Q493 – ACE2‐residues 30/31 and 34/35 in the Bp B, and hydrogen bond count in the Bp A as the most crucial descriptors. The analysis revealed that 95 % of the datasets total variance is preserved by seven out of eight calculated principle components (PC). The contribution of each PC ranges between 3.9 % and 24.1 % suggesting a meaningful selection of molecular descriptors with high variance (Table S1). The first three PCs explain over 60 % of the variance (Table S1). PC1 harbours the distance between ACE2‐residues 31–35 (or 30–34 in dogs) with 31 % contribution and S‐Q493 – ACE2‐residue 31 (or 30 in dogs) almost 27 % contribution (Figure 6, Table S1). While PC2 and PC3 share the χ1 angle of the ACE2‐F/Y82/83 as one of their main components, the lipophilic contact S‐F486 – ACE2‐F/Y82/83 (Figure 3) is strongly represented in PC2 and the deformation of the Bp C (Figure 4B) on the other hand in PC3 with contributions ranging between 23 % and 31 % (Table S1). This suggests that the molecular descriptors characterizing the Bp C and B are responsible for the majority of the variance in our data set allowing the discrimination between susceptible and non‐susceptible species (Figure S4). Furthermore, each descriptor is present as one of the main component of at least one PC. This suggests that all Bp contribute to the discrimination between susceptible and non‐susceptible species. The PCA confirms the selection of the molecular descriptors for the susceptibility discrimination obtained by visual inspection of the simulation trajectories.

### 3.6 Analysis of Squirrel, Chinese Hamster and Campbell's Dwarf Hamster ACE2 Suggests High Chance of Susceptibility to SARS‐CoV‐2

In order to prospectively validate our models, we evaluated ACE2 sequences of animals with unknown SARS‐CoV‐2 susceptibility and relevance for our environment and research. We focussed on red squirrel (*Sciurus vulgaris*) that is broadly present in urban environments in Europe and on two small hamsters (*P. campbelli*, *C. griseus*) that have been developed as a small animal COVID‐19 infection model.

We prepared red squirrel, *C. griseus*, and *P. campbelli* ACE2‐RBD complexes similarly to other species and conducted the workflow described above; (i) the depth parameters (83‐79‐SDpF) and occupancy of Bp C by RBD F486, (ii) distance plots between residues 30 (ACE2) – 417 (RBD) and 31 (ACE2) – 35 (ACE2)/31 (ACE2) – 493 (RBD) in Bp B and (iii) H‐bond counts for Bp A show similar values to that registered in human ACE2‐RBD complexes (Figures S5 and S6). Our analyses of Bp A–C indicate that the red squirrel and both small hamsters are highly susceptible to infection with SARS‐CoV‐2. These findings were supported by experiments with *P. campbelli* that have been published.^[37]^

### 3.7 Prediction of Gain‐of‐function Mutations

Finally, based on our descriptors and sequence comparisons, we suggest mutations that may render currently resistant species susceptible to SARS‐CoV‐2 infection (Table 1). This might help to establish COVID‐19 animal models for species that are broadly used in laboratories, including mice, rats, and dogs. We have investigated some of these mutants in‐silico using dynamic models.

**Table 1:** Mutations enhancing SARS‐CoV‐2 susceptibility suggested by our dynamic models.

Species	Mutation(s)			Comparable species	Rationale
	Bp A	Bp B	Bp C		
Dog	–	–	V24A	Cat	Prevents flattening of Bp C
			T81M	Human	Increases lipophilic surface of Bp C, larger side chain holds F486 in a bound position
Rat	H353K			Syrian hamster	Stabilizes H‐bond network by interaction with D38 (H‐bonds to RBD Q498)
		S27T			Lipophilic contacts to RBD F456
		N30D			Establishing of interaction to RBD K417
			I79L		Prevents narrowing of Bp C
Mouse	H353K		–	Syrian hamster	Stabilizes H‐bond network by interaction with D38 (H‐bonds to RBD Q498)
		N30D			Establishes interaction to RBD K417
		N31K			Stabilizes H‐bond network by interaction with E35 (H‐bonds to RBD Q493)

In a first step, we mutated residue 24 of canine ACE2 from valine to alanine. This polymorphism should prevent the rotation of Y82 and corresponding Bp C. The V24A mutant showed the predicted effect (Figure S7). In the 82‐78‐SDpF torsion diagram of mutated ACE2, the additional peak associated with deformation cannot be observed, which is comparable to feline RBD binding. Interestingly, a currently published cryo‐EM structure of the canine ACE2 in complex with S‐RBD (PDB‐ID: 7E3J^[35]^) confirms our observation of the Bp C deformation due to a bulky side chain of V24. The neighbouring ACE2‐residue Y82 is slightly rotated around the χ1‐angle, as predicted by our models suggesting the importance of the V24A mutation (Figure S8).

Moreover, we surmise that mutation T81M in dogs increases susceptibility to SARS‐CoV‐2 in a fashion that is similar to that conferred by human ACE2.

To test the hypothesis of narrowing the Bp C by branched side chains at position 79, we prepared and simulated an I79L mutant of rat ACE2. This mutation clearly increased Bp C occupancy and showed a wide opening of Bp C (Figure S9), which supported our hypothesis.

We further suggest mutating rat and mouse ACE2 in Bp A and Bp B to confirm stabilized RBD binding. With respect to Bp B, mutating positions 30 and 31 from neutral to charged amino acids, as is the case in Syrian hamster ACE2, might increase SARS‐CoV‐2 susceptibility of rat and mouse.

Similarly, the mutation H353K in rat and mouse ACE2 Bp A should result in a hydrogen bond network comparable to Syrian hamster protein and therefore enhancing the RBD binding. To verify this hypothesis, we prepared and simulated a mouse triple mutant (N30D, N31K, and H353K) and rat quadruple mutant (S27T, N30D, I79L, and H353K). The rat I79L single mutant showed improved Bp C binding parameters as described above, which is also the case for the quadruple rat mutant. In both species, the H353K mutation increased the hydrogen bond count in Bp A to the level of the Syrian hamster (Figure S10) confirming our hypothesis. The Bp B mutation N31K (mouse) shows higher distances between ACE2‐K31, E35 and RBD Q493 than the Syrian hamster (ca. 6 Å in mouse triple mutant versus ca. 2.5–5 Å in Syrian hamster). However, no values above 6 Å was observed as in mouse wild type and visual inspection revealed that the side chain of RBD Q493 rotates towards the binding interface suggesting enhanced ACE2‐RBD contacts. Additionally, the mutation N30D in mouse and rat shows clearly reduced distances to the partner residue in viral RBD K417 suggesting stronger ACE2 binding.

The mutation S27T in rat showed, contrary to our expectations, no significant reduction in distance distribution to RBD‐residue F456. We assumed that the more lipophilic threonine (compared to serine) would lead to closer contacts between ACE2 and RBD. Nonetheless, we suggest this mutation for experimental validation to test the results of our simulations.

## 4 Conclusions

We present mechanistic, dynamic models on an atomistic level to understand the ACE2‐SARS‐CoV‐2 S interaction in different animal species that might serve as natural reservoirs for SARS‐CoV‐2 due to frequent contacts with humans. Complementing previous studies,^[13]^ we present extensive molecular dynamics simulations that rationalize the binding of RBD to ACE2. Based on known SARS‐CoV‐2 susceptibilities in animal species and a comparison of MD trajectories, we here establish models to predict the binding of RBD to ACE2. Hence, we propose gain‐of‐function mutations for non‐susceptible species (dog, rat and mouse) that would validate our models. In addition, we predict that the red squirrel, Chinese hamster and Campbell's dwarf hamster have a high susceptibility of infection with SARS‐CoV‐2.

## Author Contribution

N.O, G.W, S.P. and T.N.N. designed the study, S.P. and T.N.N. performed and analysed the computational experiments. J.T., D.K., and N.O. provided the sequences of animal ACE2 orthologs. S.P., T.N.N., J.T., D.K., N.O., and G.W. wrote the manuscript. N.O. and G.W. supervised the study. All authors approved the final version of the manuscript.

## Conflict of interest

None declared.

5

## Supporting information

As a service to our authors and readers, this journal provides supporting information supplied by the authors. Such materials are peer reviewed and may be re‐organized for online delivery, but are not copy‐edited or typeset. Technical support issues arising from supporting information (other than missing files) should be addressed to the authors.

Supporting Information

## Data Availability

The data that supports the findings of this study are available in the supplementary material of this article.
