# Supplementary material for: ACE2‐Variants Indicate Potential SARS‐CoV‐2‐Susceptibility in Animals: A Molecular Dynamics Study
Source: Mol Inform. 2021 Aug 10;40(9):2100031. doi: 10.1002/minf.202100031 (PMC8420607; doi:10.1002/minf.202100031)
Supplement: Supplementary file 1 — Supporting Information [file MINF-40-2100031-s001.pdf]

molecular informatics

Supporting Information

ACE2-Variants Indicate Potential SARS-CoV-2-Susceptibility in Animals: A Molecular Dynamics Study

Szymon Pach[+], Trung Ngoc Nguyen[+], Jakob Trimpert, Dusan Kunec, Nikolaus Osterrieder,* and Gerhard Wolber*© 2021 The Authors. Molecular Informatics published by Wiley-VCH GmbH. This is an open access article under the terms of the Creative Commons Attribution License, which permits use, distribution and reproduction in any medium, provided the original work is properly cited.

Supporting Information

ACE2-Variants Indicate Potential SARS-CoV-2-Susceptibility in Animals: An Extensive Molecular Dynamics Study

Szymon Pach†[a], Trung Ngoc Nguyen†[a], Jakob Trimpert[b], Dusan Kunec[b], Nikolaus Osterrieder*[b], Gerhard Wolber*[a]

ORCID IDs: Szymon Pach: 0000-0001-6109-7123, Trung Ngoc Nguyen: 0000-0002-7415-4390, Jakob Trimpert: 0000-0003-1616-0810, Dusan Kunec: 0000-0001-6697-7621, Nikolaus Osterrieder: 0000-0002-5313-2176, Gerhard Wolber: 0000-0002-5344-0048

---

[a] *Pharmaceutical and Medicinal Chemistry, Institute of Pharmacy, Freie Universität Berlin*
Königin-Luise-Str. 2 - 4, 14195 Berlin, Germany

[b] *Institut für Virologie, Freie Universität Berlin*
Robert-Von-Ostertag-Str. 7 - 13, 14163 Berlin, Germany
**e-mail: Gerhard Wolber (gerhard.wolber@fu-berlin.de), Nikolaus Osterrieder (no.34@fu-berlin.de)*

[†] *These authors contributed equally.*

Table S1: Principle component (PC) analysis of eight selected molecular descriptors.

**Feature**	**Eigenvalue**	**Variance [%]**
PC 1	1.93	24.1
PC 2	1.67	20.8
PC 3	1.27	15.9
PC 4	1.07	13.3
PC 5	0.74	9.2
PC 6	0.62	7.8
PC 7	0.40	5.0
PC 8	0.32	3.9

		Bp C				Bp B		Bp A
**Feature**	**Depth**	**Defor-mation**	**χ1 Angle ACE2-F/Y82/83**	**Lipophilic contacts S-F486 – ACE2-F/Y82/83**	**Distance S-Q493 – ACE2-residue 30/31**	**Distance ACE2-residues 30/31 – 34/35**	**Distance S-K417 – ACE2-residue 29/30**	**Hydrogen bond count**
PC 1	12,9%	2,4%	6,5%	5,5%	26,9%	31,0%	6,5%	8,2%
PC 2	18,8%	0,1%	24,5%	23,2%	2,7%	0,5%	13,6%	16,6%
PC 3	0,1%	30,6%	24,0%	1,3%	16,5%	7,8%	8,7%	10,9%
PC 4	21,2%	44,5%	0,0%	0,2%	2,3%	7,2%	20,1%	4,4%
PC 5	13,1%	0,5%	1,2%	64,1%	4,1%	4,3%	6,7%	6,0%
PC 6	0,4%	1,8%	0,0%	0,1%	0,0%	0,8%	43,1%	53,8%
PC 7	6,6%	12,9%	18,0%	0,5%	26,8%	34,2%	0,8%	0,2%
PC 8	26,9%	7,1%	25,7%	5,2%	20,6%	14,1%	0,5%	0,0%

Abbreviations: ACE2- Angiotensin-converting enzyme 2, Bp- binding pocket, PC- principle component, S- spike. Red numbers represent the highest value in a row, the orange numbers the second-highest value.

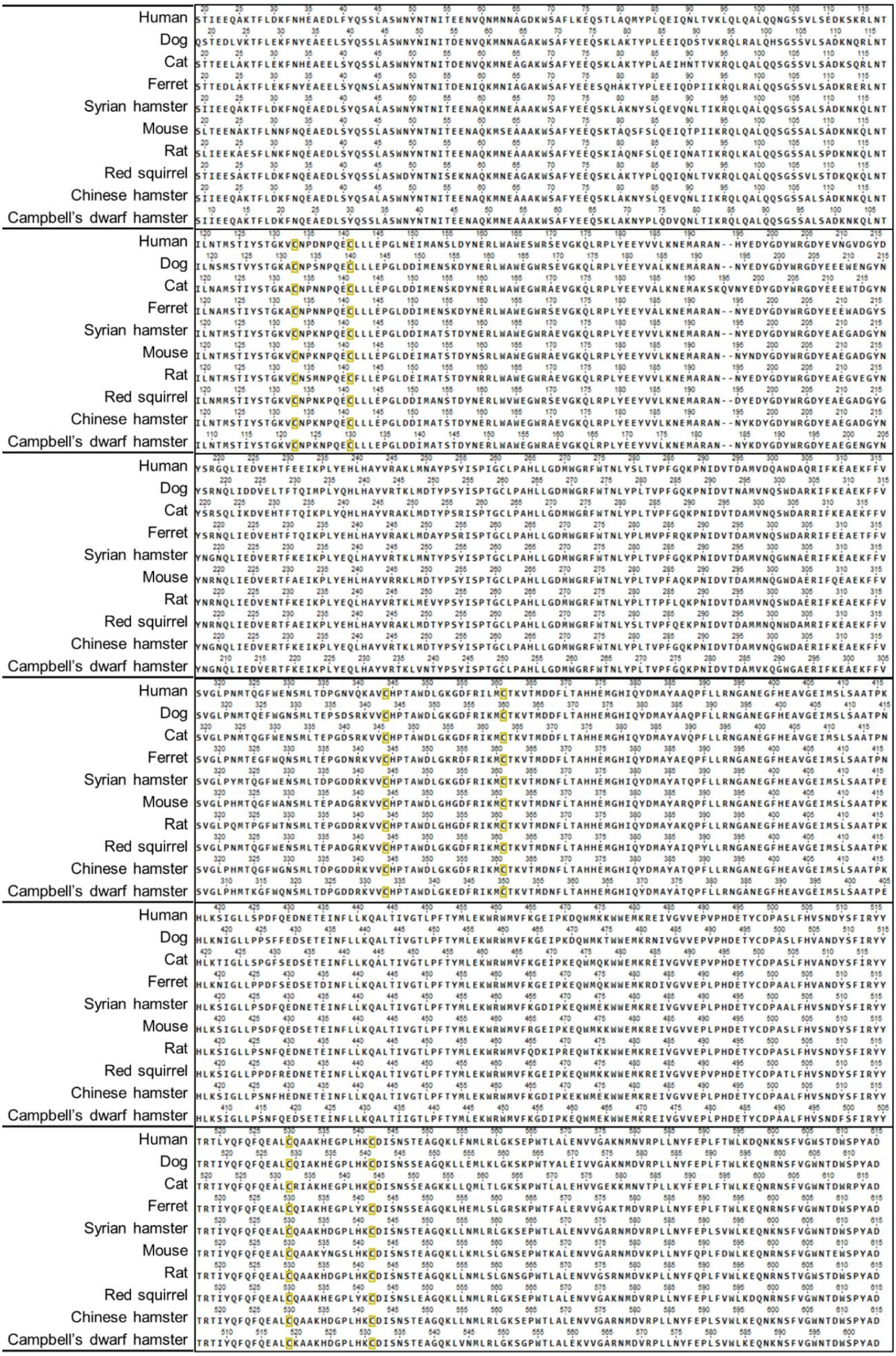

Figure S1: Sequence alignment of human and animal angiotensin-converting enzyme 2 performed with MOE 2019.0102 (Molecular Operating Environment, Chemical Computing Group ULC, Montreal, Canada). Colour code: yellow box- cysteine residues involved in a disulfide bridge.

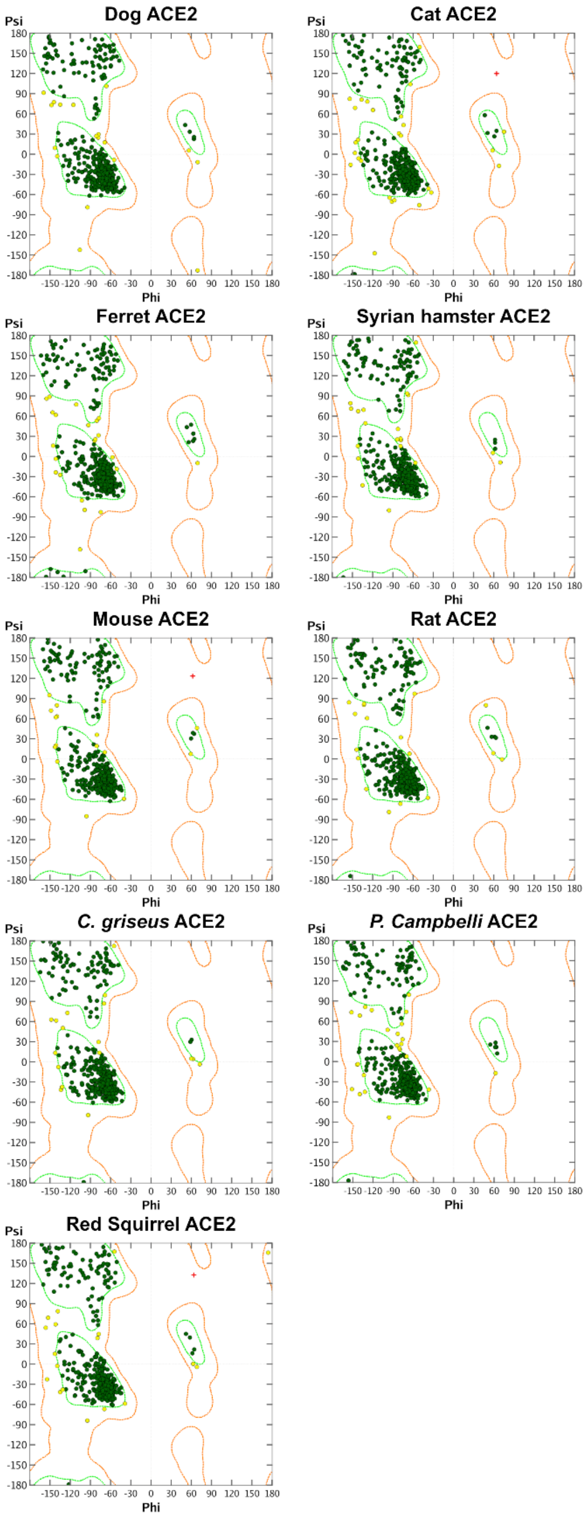

Figure S2: Ramachandran plots for all ACE2 homology models. Symbol code: green point- residue with favourable geometry, yellow point- residue with allowed geometry, red cross- outlier. The figure was generated using MOE 2019.0102 (Molecular Operating Environment, Chemical Computing Group ULC, Montreal, Canada).

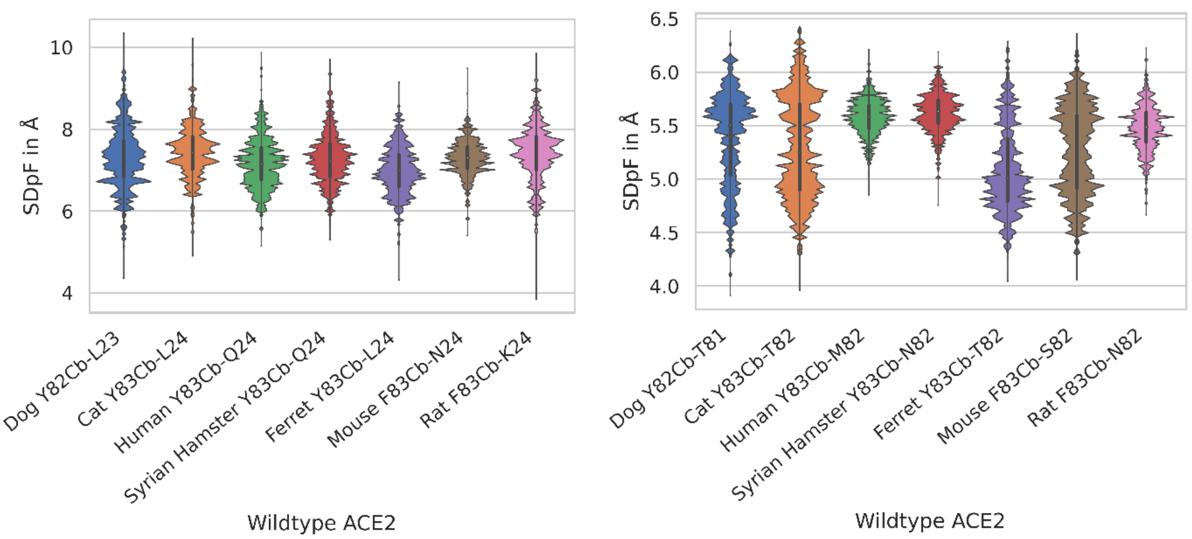

Figure S3: Distance distribution between Bp C key residues for analyzed wildtype ACE2 species. Shortest Distance per Frame (SDpF) between the Cb atom of residue 83 (or 82 in dog) and side chains flanking the binding pocket C (residues 24 and 82 or 23 and 81 in dog) representing the ‘depth’ of Bp C.

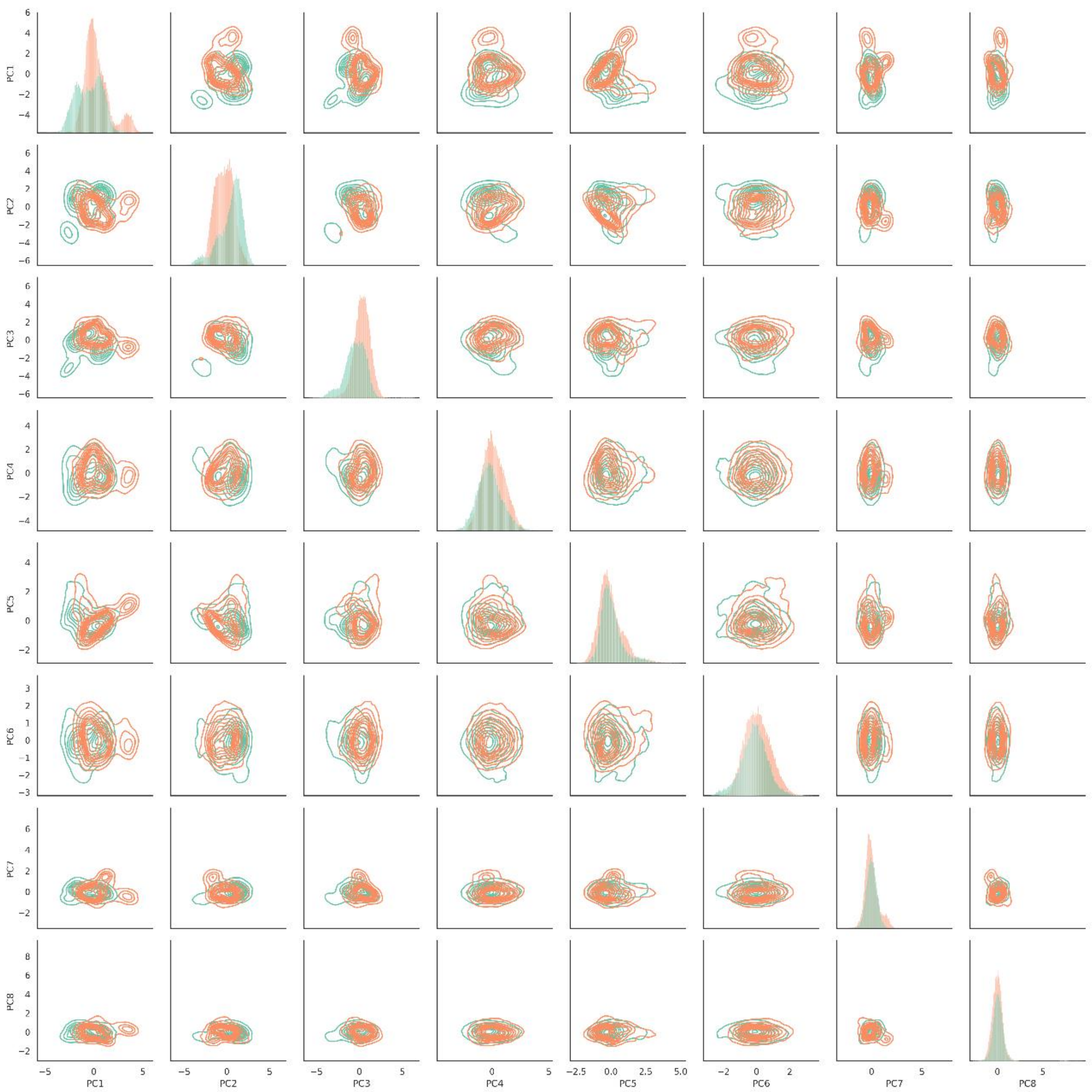

Figure S4: Histograms of principle components (PC) and kernel density plots of PC pairs visualizing the variance dependent ability of our feature set to discriminative between susceptible (orange lines) and non-susceptible species (green lines).

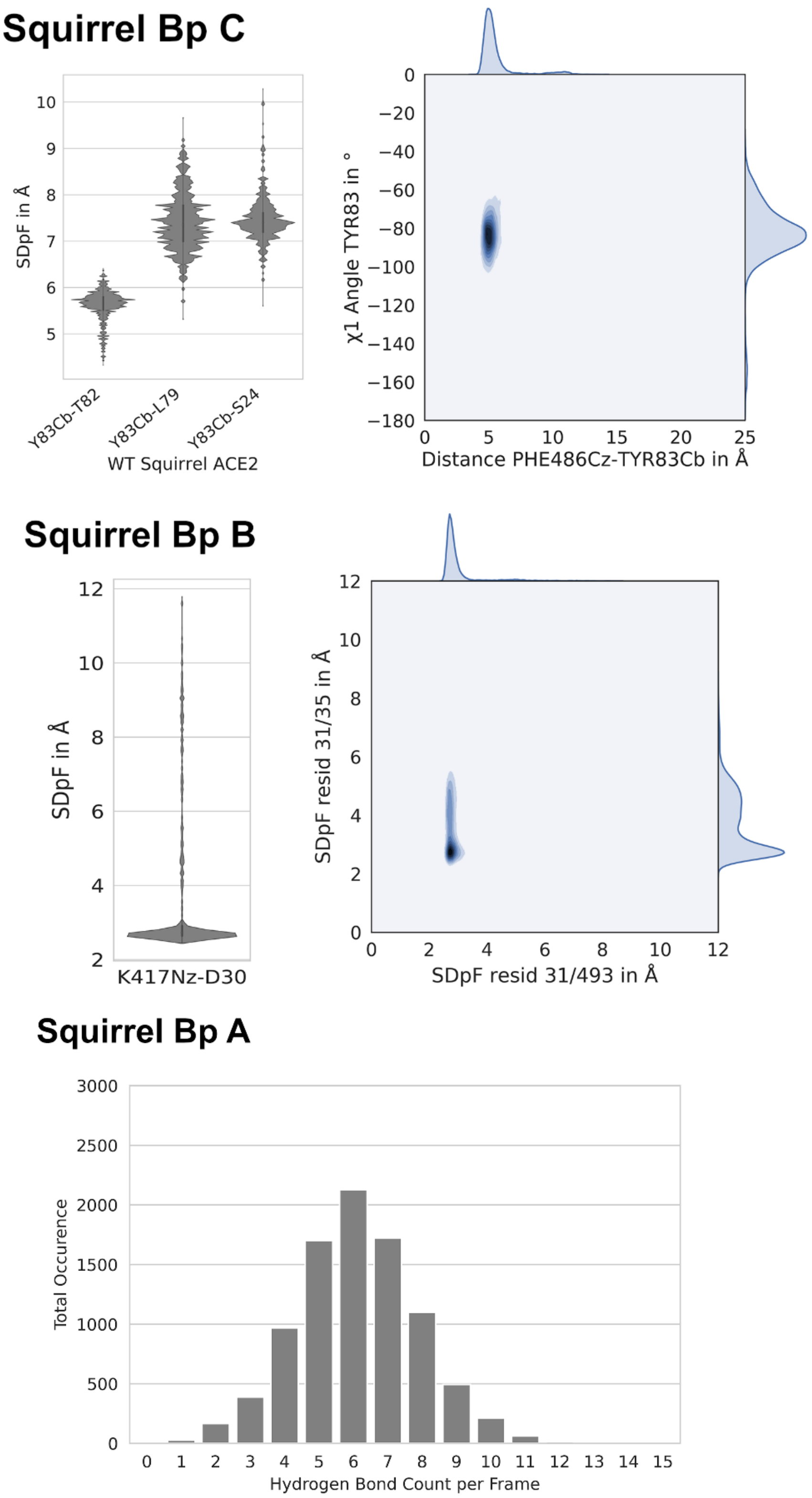

Figure S5. Descriptors calculated for red squirrel ACE2-RBD simulations. **Bp C**: Shortest Distance per Frame (SDpF) between Cb atom of Y83 and side chains of residues flanking the binding pocket; **Bp B**: Distance distribution for interactions between Nz atom of RBD K417 and the side chain of ACE2 residue 30, and kernel density plots summarizing the interactions between ACE2-residue 31 and RBD residue Q493; **Bp A**: histograms representing the total occurrence of hydrogen bonds in binding pocket A between ACE2 residues 37, 38, 41, 42, 353, 355 and RBD residues 449, 496, 498, 500, 501, 505.

C. griseus Bp C

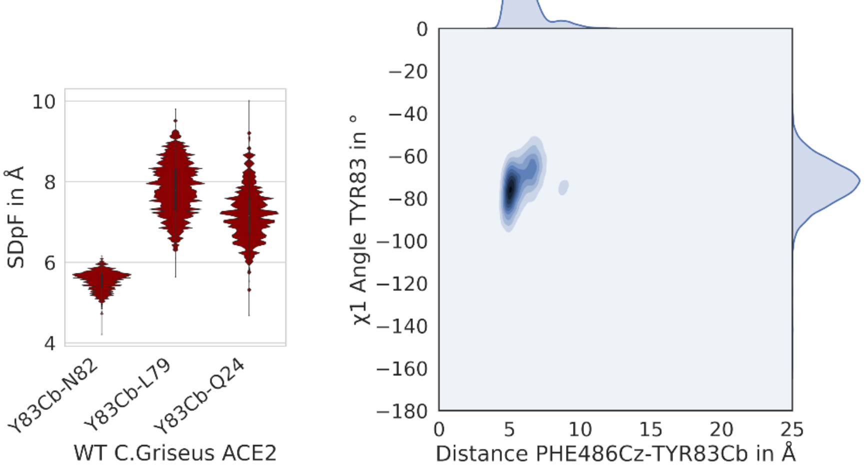

C. griseus Bp B

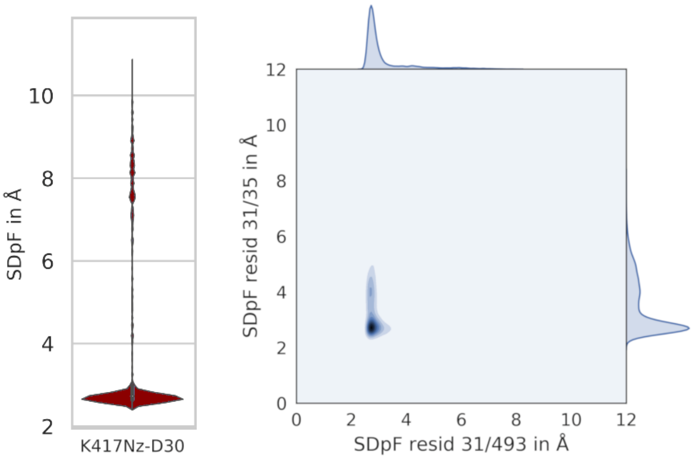

C. griseus Bp A

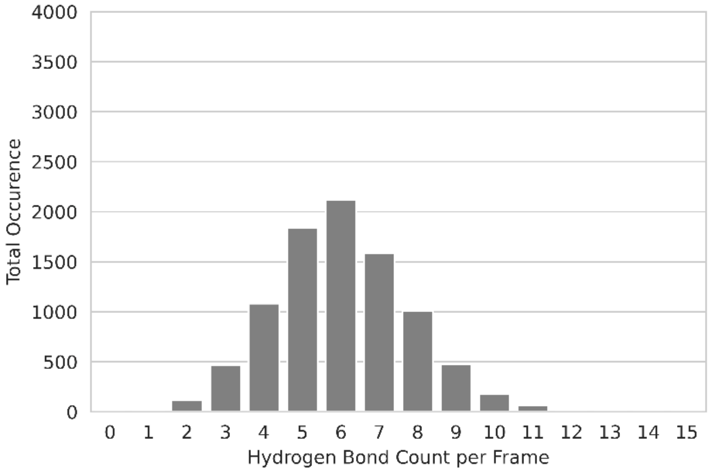

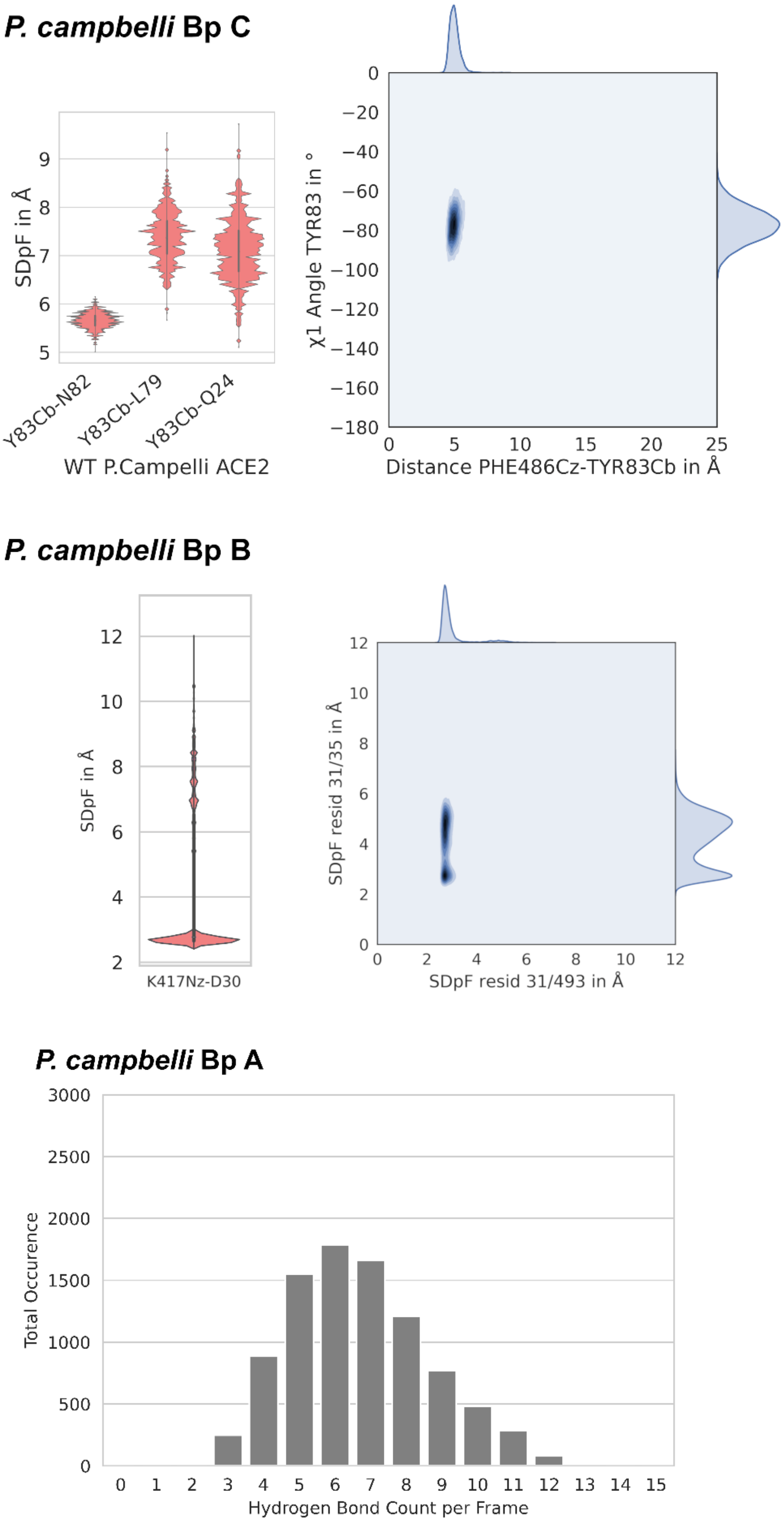

Figure S6. Descriptors calculated for small hamsters (*C. griseus*, *P. campbelli*) ACE2-RBD simulations. **Bp C**: Shortest Distance per Frame (SDpF) between Cb atom of Y83 and side chains of residues flanking the binding pocket; **Bp B**: Distance distribution for interactions between Nz atom of RBD K417 and the side chain of ACE2 residue 30, and kernel density plots summarizing the interactions between ACE2-residue 31 and RBD residue Q493; **Bp A**: histograms representing the total occurrence of hydrogen bonds in binding pocket A between ACE2 residues 37, 38, 41, 42, 353, 355 and RBD residues 449, 496, 498, 500, 501, 505.

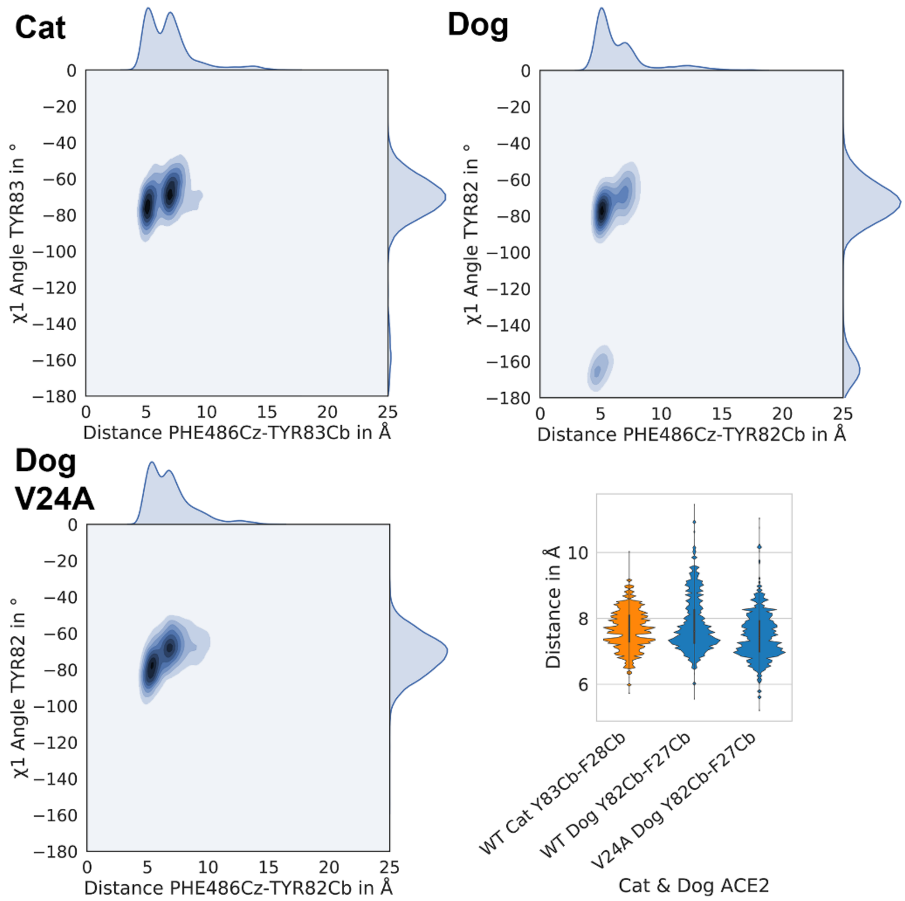

Figure S7. Occupation of binding pocket C indicated by kernel density plots (x-axis: distance F486 Cz - Y82 Cb; y-axis: χ1 angle of central residue 82/83) and Bp C deformation of in dog ACE2 wild type, V24A mutant, and homologous residue 83 in cat (Y83/82 Cb – F28/27 Cb distance).

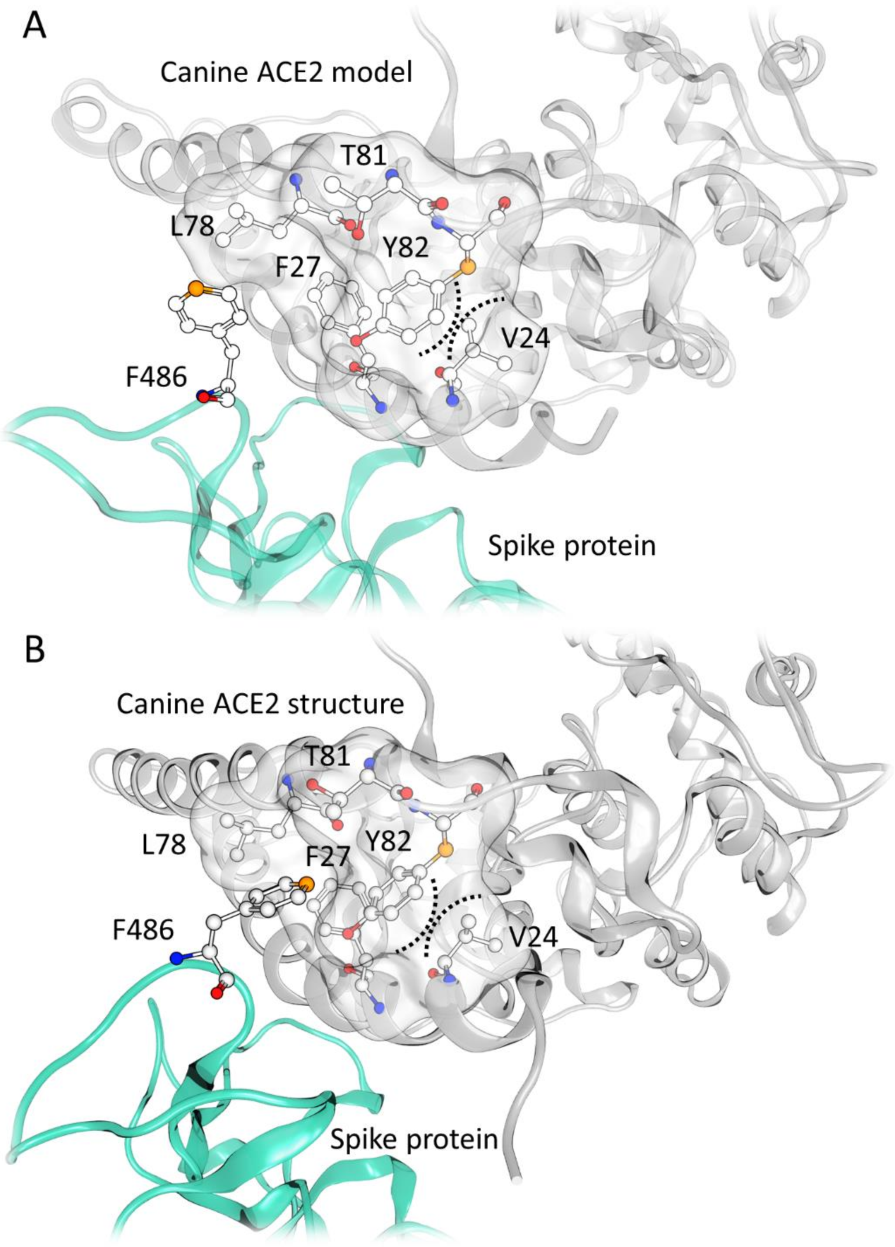

Figure S8. Comparison of (A) the "fixed state" in our model of canine angiotensin-converting enzyme 2 (ACE2) in complex with viral spike protein extracted from a molecular dynamics simulation and (B) the cryoscopic electron microscopy structure of the dog ACE2 in complex with the viral spike protein.

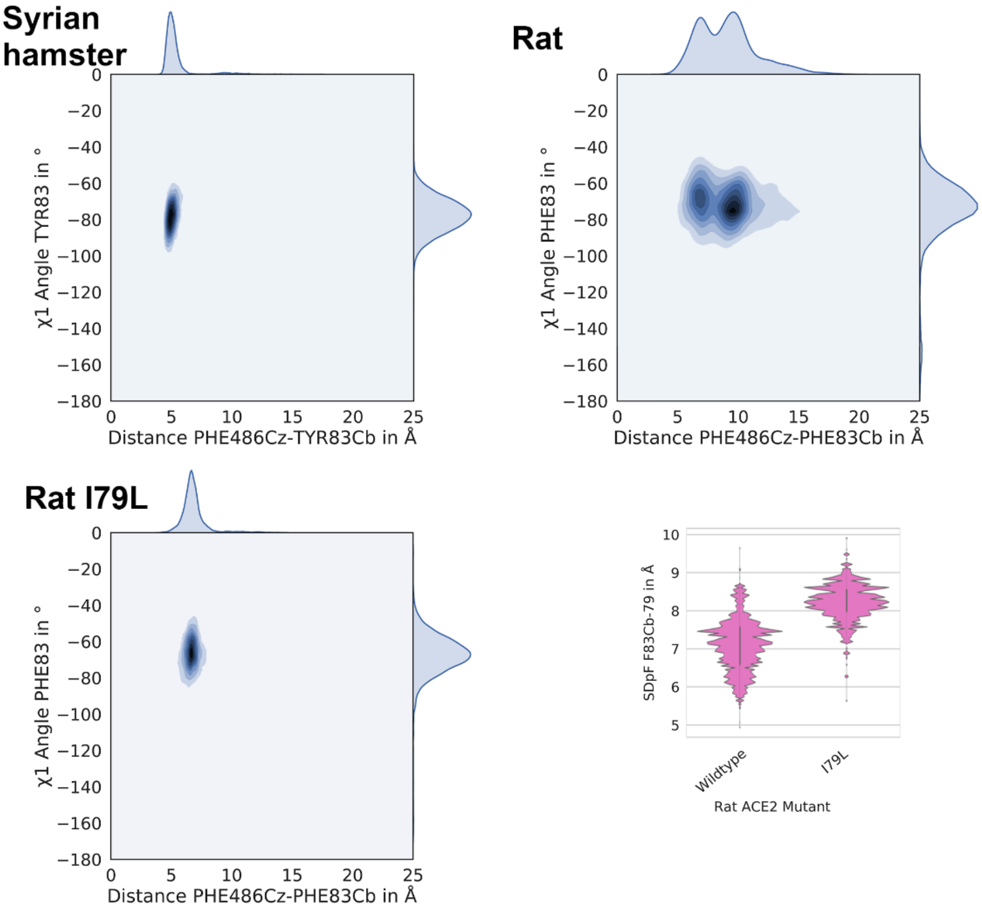

Figure S9. Kernel density plots summarizing the occupation of binding pocket C (x-axis, surrogate parameter: distance F486 Cz - Y83 Cb) and rotamers of central residue 82 (or 83 in cats). The lower right panel shows the opening of the Bp C based on the SDpF-83-79 (Shortest Distance per Frame) parameter.

Mouse 3-fold Mutant Bp C

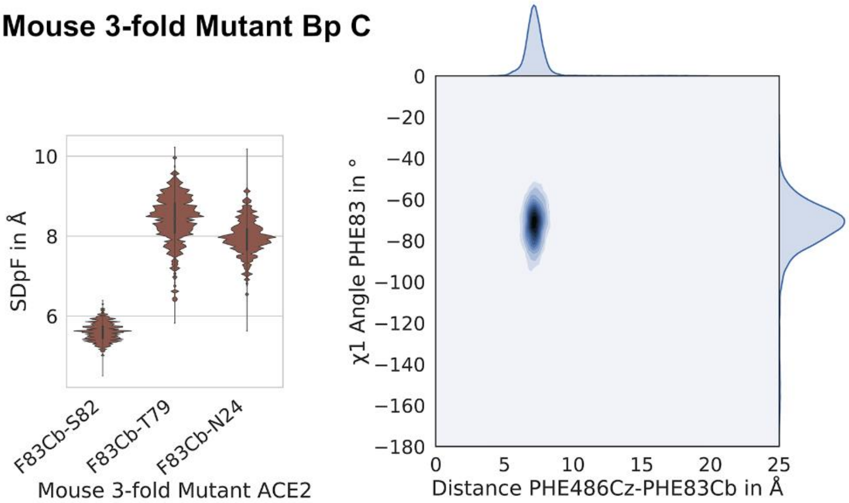

Mouse 3-fold Mutant Bp B

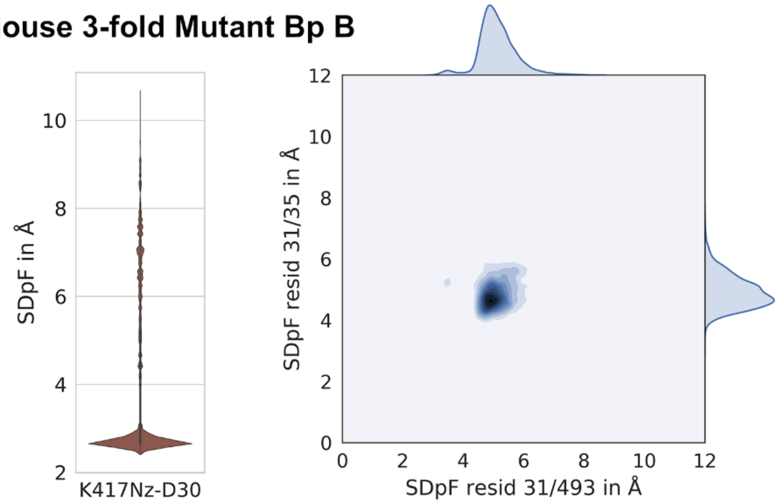

Mouse 3-fold Mutant Bp A

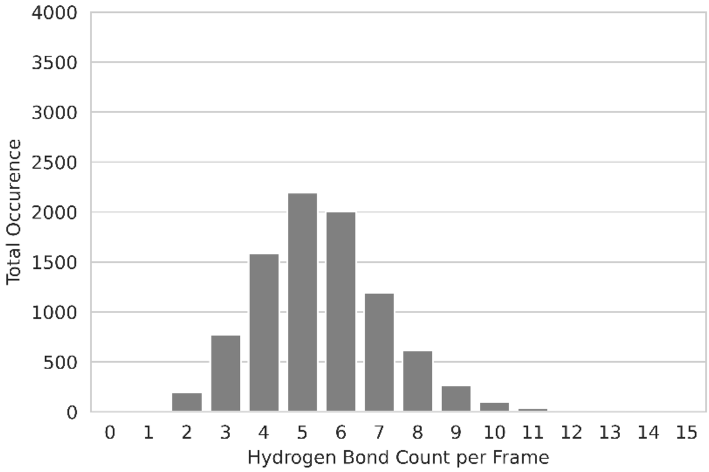

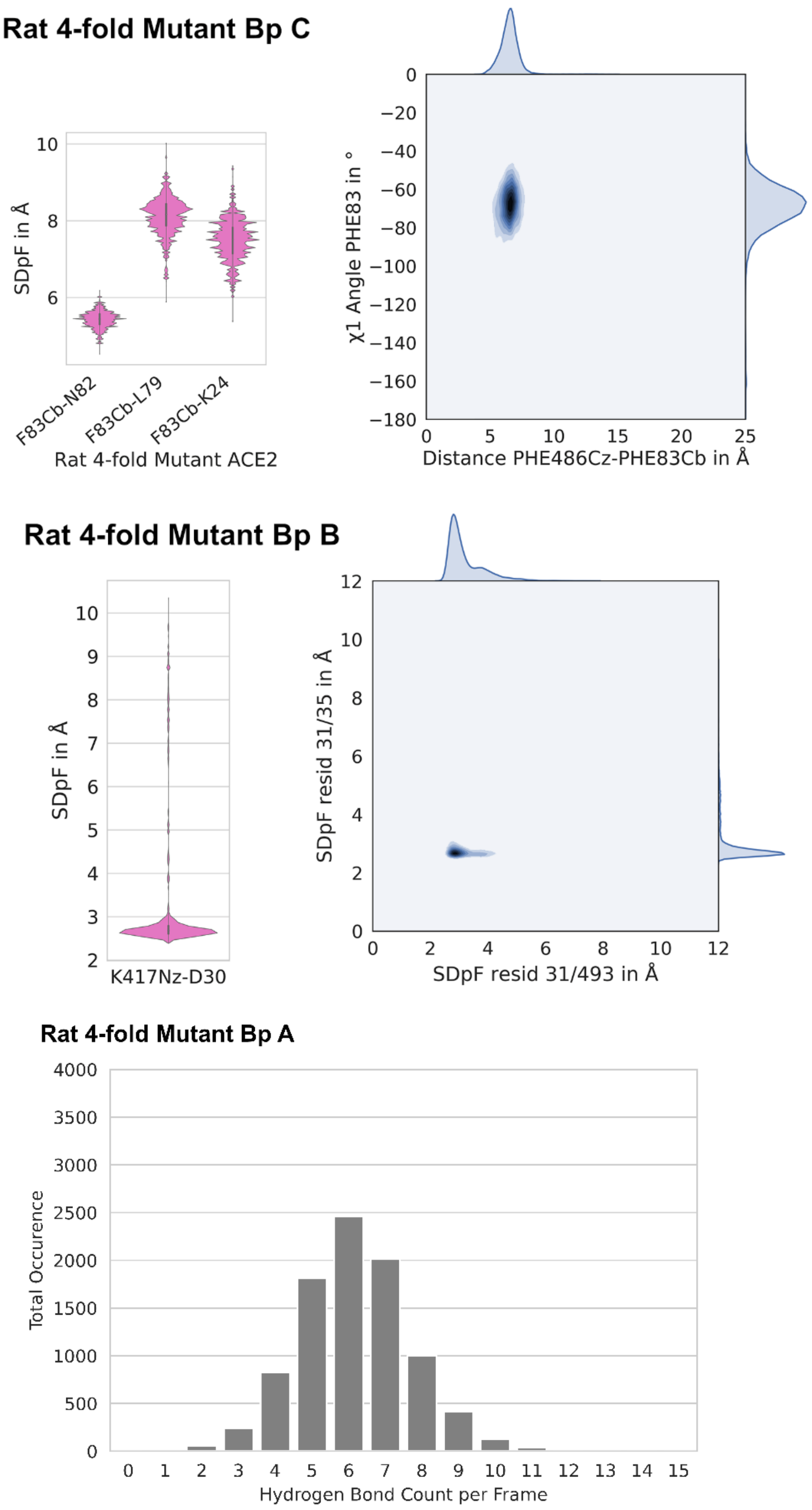

Figure S10. Descriptors calculated for mouse triple mutant (upper panels) and rat quadruple mutant (lower panels) ACE2-RBD simulations. **Bp C**: Shortest Distance per Frame (SDpF) between Cb atom of Y83 and side chains of residues flanking the binding pocket; **Bp B**: Distance distribution for interactions between Nz atom of RBD K417 and the side chain of ACE2 residue 30, and kernel density plots summarizing the interactions between ACE2-residue 31 and RBD residue Q493; **Bp A**: histograms representing the total occurrence of hydrogen bonds in binding pocket A between ACE2 residues 37, 38, 41, 42, 353, 355 and RBD residues 449, 496, 498, 500, 501, 505.
